# Distinguishing Latent from Active *Mycobacterium tuberculosis* Infection Using Elispot Assays: Looking Beyond Interferon-gamma

**DOI:** 10.3390/cells1020089

**Published:** 2012-05-07

**Authors:** Camilla Tincati, Amedeo J. Cappione III, Jennifer E. Snyder-Cappione

**Affiliations:** 1 Department of Medicine, Surgery, and Dentistry, Clinic of Infectious Diseases and Tropical Medicine, San Paolo Hospital, University of Milan, 20142 Milan, Italy; Email: camilla.tincati@unimi.it; 2 EMD Millipore Corporation, 17 Cherry Hill Drive, Danvers, MA 01923, USA; Email: amedeo.cappione@merckgroup.com; 3 Department of Microbiology and Flow Cytometry Core Facility, Boston University School of Medicine, Boston, MA 02118, USA

**Keywords:** mycobacterium tuberculosis, Elispot, polyfunctional T cell, IFN-gamma, LTBI, active MTB

## Abstract

*Mycobacterium tuberculosis* (MTB) is a global heath epidemic, its threat amplified by HIV infection and the emergence of multidrug-resistant tuberculosis (MDR-TB). Interferon (IFN)-gamma release assays (IGRAs) have improved the accuracy of detection of MTB exposure in some subject groups as compared to the Tuberculin Skin Test (TST). However, as IFN-gamma is produced by both fully rested and more recently activated populations of memory T cells, it is not surprising that the measurement of this cytokine alone cannot accurately distinguish Latent TB Infected (LTBI) subjects from those with active (infectious) disease. Accurate and rapid diagnosis of infectious individuals would allow medication to be properly allocated and other actions taken to more effectively curtail MTB spread. Analysis of multi-cytokine profiles *ex vivo* after stimulation of PBMCs from LTBI and active MTB subjects indicate the real possibility of successfully discerning these two disease states within 24 hours of a subject’s blood draw. Due to the unparalleled sensitivity, low cost, and ease of use of Elispot assays, we propose that via a multiplex Elispot platform the accurate distinction of LTBI from active MTB-infected individuals is within reach.

## Abbreviations

MTB: mycobacterium tuberculosis
MDR-TB: multi-drug resistant tuberculosis
IGRAs: IFN-gamma release assays
LTBI: latent TB infected
IGRA: IFN-gamma release assay
PBMC: Peripheral Blood Mononuclear Cells
BCG: Bacille Calmette Guerin
RD1: region of deletion 1

## 1. Introduction

*Mycobacterium tuberculosis* (MTB) is a global health crisis, with one in three individuals predicted to have been exposed to MTB worldwide. Of those exposed, eight million develop symptoms and approximately two million succumb to the infection each year. The predominance of HIV and MTB co-infection in many developing countries and the emergence of drug resistant MTB strains have contributed to these staggering global statistics [1]. 

After MTB exposure, usually via inhalation, lung macrophages are infected, spurring inflammation and an adaptive immune response. MTB-antigen-specific immunity usually successfully controls the pathogen, although viable bacteria do remain in granulomas for extended periods of time [2]; this is known as latent TB infection (LTBI). Most LTBI individuals are asymptomatic for their lifetime, with only approximately 15% ever developing active disease [3]. However, in HIV-infected individuals, there is a 5–10% risk of conversion from LTBI to active disease each year [3]. It is those with active MTB that are most likely to spread the disease and pose the largest health risk to the community.

The only effective vaccine to date is BCG (Bacille Calmette Guerin), which limits pediatric MTB but is not as effective in preventing adult pulmonary disease [4]. Also, treatment for MTB has not changed significantly since 1976 [3]. With a marginally effective vaccine and no new treatments on the horizon, a new sensitive and rapid method to distinguish LTBI from active (infectious) disease would allow quick treatment and containment of infectious individuals, limiting MTB spread and mortality.

## 2. Current Methods to Detect MTB Exposure/Infection

MTB infection is traditionally and still widely diagnosed using the Tuberculin Skin Test (TST). In this method, an individual is injected with Purified Protein Derivative (PPD) and evidence of antigen-specific immune memory is assessed via swelling of the injected site within 48 to 72 hours. The simplicity and ease of readout are clear advantages; however, TST often provides ambiguous results in immuno-suppressed individuals [3], and those never exposed to MTB but previously vaccinated with BCG can present with positive test results. 

In the last decade, new assays were FDA approved to diagnose MTB in the United States: the TSPOT™.TB (Oxford Immunotec), the QuantiFERON^®^-TB Gold (QFT), and the QuantiFERON^®^-TB Gold In-Tube (QFT-IT) (Cellestis Limited). In the TSPOT™.TB and QFT, blood is drawn from the subject and IFN-gamma is measured after stimulation of PBMC with Early Secreted Antigen Target (ESAT-6) and Culture Filtrate Protein (CFP-10) antigens, both found within the RD1 (Region of Deletion 1) genomic segment of MTB. The QFT-IT includes the TB7.7p4 peptide coated to vacutainer tubes for cell stimulation without the need for cell isolation. These Elispot or ELISA-based methods, collectively referred to as IFN-gamma-release assays (IGRAs), offer noted advantages in the detection of latent MTB infection over TSTs. IGRAs demonstrate high specificity for MTB and, unlike the TST, are far less likely to give positive results from individuals who are BCG vaccinated and never MTB exposed. Also, the sensitivity of IGRAs increases the likelihood of detecting MTB infection in immuno-suppressed subjects, such as those who are HIV+ and are most likely to progress to active infection [5]. IGRAs clinical use is most noted due to its strong predictive value to active infection amongst MTB contacts [6] Another cytokine, IP-10, performs similar to the IGRAs for MTB diagnosis and positivity rates were less dependent on CD4 counts in HIV+ subjects, implicating this analyte as one of choice for detection of TB in immuno-suppressed individuals [7]. Both the IGRAs and IP-10 release assays have improved the ability to detect MTB exposure as compared with TST; however, they cannot reliably distinguish between latent and active MTB infection [8,9]. Also, analysis of a large range of transcriptional biomarkers in whole blood revealed signatures of actively infected subjects that correlate with the severity of their radiological disease; some LTBI and actively infected patients were not distinguishable using this platform [10]. There is currently no standard test to determine latent (LTBI) from active (infectious) MTB. 

## 3. Limitations of IFN-gamma as the Sole Measurement of Immune Status

When a naïve T cell encounters peptide antigen and MHC, it will differentiate into an antigen-experienced (memory) T cell. Depending on the cytokine milieu and other factors in the microenvironment at priming, T cells can differentiate into Type 1, Type 2, or other functional lineages, such as Th17 [11]. While most immune responses include Th1/Tc1 cells (the CD4 and CD8 cells of the Type 1 lineage, respectively), and many of the T cells within that lineage secrete IFN-gamma upon re-stimulation *ex vivo*, these cells likely represent only one fraction of the composite T cell response induced by a pathogen or vaccine. Also, not all Th1/Tc1 cells secrete IFN-gamma upon re-stimulation [12,13]. In addition, other functions commonly associated with Type 1 T cells, such as cytotoxicity, are regulated and expressed independently of IFN-gamma in CD8 T cell populations [12,14,15]. Therefore, the numbers of IFN-gamma-producing cells can be a poor correlate of cytotoxic or “CTL” frequencies in one’s experimental system or patient sample. However, in many vaccine trials, infectious disease studies, and in the IGRAs, IFN-gamma is the sole immunological readout and therefore a considerable and important proportion of the immune response is not considered. 

Furthermore, as IFN-gamma can be rapidly produced by recently activated effector or fully rested memory T cells, measurement of only this one analyte will likely reveal very little about the state of infection/disease in an individual. The IFN-gamma response after ESAT-6 or CFP-10 stimulation are different in MTB subjects in different states of disease when measured *ex vivo* in one study [16] but not another [17]; this is consistent with the findings in other infections such as HIV, where there is no consistent association with peptide-specific IFN-gamma secretion and viral load [18]. 

Therefore, while IFN-gamma measurement can serve as a useful biomarker of previous antigen encounter, its value when measured alone at determining disease state or vaccine efficacy is often limited and can be very misleading. Multiple effector functions secreted by the composite MTB-specific T cell memory pool must be simultaneously measured to perform accurate T cell diagnostics of LTBI *versus* active MTB disease. 

## 4. Cytokine Analysis of the *ex Vivo* MTB-specific T Cell Response in LTBI, Active MTB Subjects

In recent years, analysis of cytokines other than IFN-gamma and also multiple effector functions in combination from the MTB-specific immune response of LTBIs and active MTB subjects were compared. Some interesting trends are emerging to suggest that a functional bio-signature for LTBI subjects, distinct from active infected individuals, is attainable.

### 4.1. IL-2, IFN-gamma, TNF-alpha, and the “Polyfunctional” T Cell

Flow cytometric analysis allows the simultaneous measurement of multiple cytokines from individual T cells after short-term stimulation with MTB antigens. Comparison of the percentages of T cells secreting all possible combinations of IL-2, IFN-gamma, and TNF-alpha after ESAT-6 or CFP-10 stimulation of PBMC from individuals with LTBI *versus* active MTB was performed. Higher percentages of CD4+ T cells that are “polyfunctional” triple producers (IL-2+, IFN-gamma+, TNF-alpha+) were found in the PBMC of LTBIs compared with active MTB subjects [17,19], with statistical significance in one study [17]. Also, longitudinal analysis of MTB-specific T cell responses from subjects with active disease (before and after treatment) found that the proportion of IL-2+, IFN-gamma+, TNF-alpha+ triple producing cells was higher, on average, after treatment [17,19]. Measurement of MTB-specific single-positive TNF-α+ CD4 T cells showed the best discriminatory potential for these two disease states in one study [17]. Also, there is a positive correlation of *ex vivo* proliferative capacity of MTB-specific T cells and the number of TNF-alpha+ IL-2+ IFN-gamma+ cells after MTB antigenic stimulation [19]. Similar to this flow cytometric data, two-color fluorescent Elispot assays for IFN-gamma and IL-2 found more dual producing cells in the LTBIs compared with active TB subjects; furthermore, this signature of IFN-gamma only cells in untreated MTB subjects with active disease shifted to dual IFN-gamma+ IL-2+ T cells in the months after treatment was initiated [20]. Taken together, these data suggest that similar to what is found in other infectious diseases [21,22], high bacterial load is congruent with functional and proliferative exhaustion of antigen-specific T cells, with MTB-specific T cells likely returning to a resting state after treatment and re-acquiring the ability to secrete multiple cytokines and proliferate when antigen is re-encountered.

While analysis of TNF-alpha and IL-2 in addition to IFN-gamma shows great potential to conclusively distinguish LTBI from active MTB subjects, either no differences in the profile of the IL-2+, IFN-gamma+, TNF-alpha+ responses to ESAT-6 or CFP-10 between active and LTBI subjects [23] or that the IL-2+, IFN-gamma+, TNF-alpha+ T cell frequencies were actually higher in the active TB subjects [24] has been reported as well. Use of recombinant proteins in re-stimulation cultures, length and nature of stimulation assays, and differences in patient cohorts and number of events collected in flow cytometric assays could account for why these results differ from other findings. Alternatively, it is possible that T cell functions from other effector lineages must be measured to reliably determine LTBI *versus* active MTB disease. 

### 4.2. IL17

IL17 is a cytokine associated with inflammation and anti-viral responses, and is produced by the Th17 lineage of memory T cells. In MTB infection, the frequency of IL17-producing CD4+ T cells after PMA and Ionomycin stimulation was significantly lower in active MTB subjects compared to both LTBI and healthy donors [25]; the frequency of IL17+ T cells after BCG stimulation was also found to be lower in active MTB subjects compared with healthy controls, and IL17 was produced by T cells within PBMC of MTB subjects after ESAT-6/CFP 10 peptide stimulation [26] Also, IL-17 production by CD4+ T cells in response to CFP-10/ESAT-6 peptides was detected from pleural fluid samples from subjects with tuberculous pleurisy (TBP) [27]. This cytokine therefore may prove an important component of anti-MTB immunity and useful for detection of LBTI *versus* active MTB disease states. 

### 4.3. Type 2 Cytokines/ T regulatory cells (Tregs)

While Th1 cytokines (IFN-gamma, TNF-alpha) and the Th17 cytokine IL-17 are well established as integral components of a successful immune response to infection, these functions can cause significant immuno-pathology to the host. Therefore, T cell effector functions with immunosuppressive roles, such as IL-4, IL-5, IL-10, IL-13, TGF-beta and the direct T cell inhibitory T regulatory cells (Tregs), are often a substantial and integral component of an antigen-specific immune response. TGF-beta and IL-10 are produced in response to PPD from PBMC of infected subjects *ex vivo* [28]. Cytometric Bead Array assays of culture supernatants after PBMC stimulation in Quantiferon^®^ Gold In-Tube tests reveal significantly more IL-4 production from the PBMC of active compared with latent TB subjects, with IFN-gamma not significantly different between the two groups [29]. Also, 20–25% of pulmonary TB patients with newly active disease present a diminished TST response [30]; these negative TSTs associate with lower IFN-gamma T cell responses to PPD in *ex vivo* re-stimulation assays [28,31]. Negative TST results also routinely appear in subjects with miliary TB and other extra pulmonary forms of disease, with reversion to positive responses after MTB treatment [30]. IL-10 and TGF-beta are elevated in active MTB subjects and associated with suppression of MTB-specific immune responses [28]. Also, FOXP3+ CD4 T cells are higher in frequency in pleural fluid compared with peripheral blood [25]; these findings implicate a role of Tregs at the site of MTB replication. The relative Treg frequencies in the PBMC of LTBI and active MTB subjects are not yet clear. 

This immunosuppressive arm of the immune response may arise to dampen the inflammation and reduce immuno-pathology during onset of active disease, or alternatively, MTB may have evolved to induce type 2 responses to inhibit autophagy in macrophages, as IFN-gamma promotes while IL-4 and IL-13 directly inhibit this process [32]. Regardless of the cause, the apparent emergence of these Type 2 cytokines render them attractive candidates for analysis when seeking to define bio-signatures of LTBI and active MTB disease states for immunodiagnostics. 

## 5. The Power of a Multiplexed Elispot Platform to Distinguish LTBI from Active MTB Disease

Taken together, the findings of Th1, Th2, Th17, and other effector functions in LTBI as compared with active MTB subjects indicate that combinational analysis of multiple functions will likely provide a cytokine bio-signature that will conclusively distinguish these two states of infection. Elispot assays present clear advantages over other methods currently used for analysis of *ex vivo* T cell effector functions, and we propose Elispots are the platform of choice to distinguish LTBI *versus* active MTB infection. 

### 5.1. Elispot *Versus* Flow Cytometry (Intracellular Cytokine Staining)

Elispots, like flow cytometry-based Intracellular Cytokine Staining (ICS), can directly determine the frequency of immune cells responding to a particular antigen, a core competency for immune diagnostics. In fact, while the putative naïve T cell repertoire is comprised of a vast number (≥10^12^) of TCR specificities, for any single infective agent, the frequency of circulating antigen-specific memory cells is quite low, typically in the range of 1:10,000–1,000,000. While capable of single-cell resolution, detection of this rare cell pool can be a challenge for flow cytometry, whose lower limit of sensitivity for identifying functional T cell frequencies is reported to be 0.02% [33]. By contrast, in routine 96-well Elispot assays, limits of 0.00025% (1 spot forming unit per 400,000 input PBMC) are commonly observed [34]; this equates to a near 100-fold greater detection sensitivity compared with flow cytometry. In fact, the Elispot assay has demonstrated signal linearity for cultures in the range of 1 × 10^6^ PBMC [35]; thus, the only true constraint on this technique appears to be the number of cells available for testing, size can also be increased to accommodate larger cell numbers. Also, Elispots are currently one of the only techniques permitting quantification of secretory activity at the single cell level. Since detection by ICS occurs prior to cytokine release, there is the potential for misleading results due to post-translational modulation of protein expression [36]. Further, while both ICS and Elispots measure cytokine secretion independent of kinetics within the culture period, a significant fact given the unsynchronized nature of the responding T cell pool, the duration of the ICS assay is limited by the toxicity of protein transport inhibitors. Therefore, detection of some cytokines may not be possible when ICS is used.

### 5.2. Elispot *Versus* Supernatant Analysis (ELISA, Multiplex Bead Array Technology)

While ELISAs and bead-based measurement of cytokines from culture supernatants offer some advantages [37], there are limitations that preclude their acceptance as a T cell diagnostic platform for MTB disease states. For example, bulk supernatant assays lack the sensitivity for extremely low frequency signal detection. Spot size gating of Elispots using quantitative image analysis platforms permits a clear distinction between the diagnostically relevant large T cell-derived spots and smaller non-specific bystander spots. Since bulk assays cannot provide information on a ‘per cell’ basis, the contribution of Ag-specific responses may be diluted by background from the innate immune system, resulting in overall signal flattening; this issue is most relevant in PBMC cultures where the Ag-specific T cell pool is vastly outnumbered. The sensitivity threshold for cytokine measurements in culture supernatants is further diminished in these assays by analyte dilution in the surrounding milieu, absorption by bystander cells, and enzymatic degradation. 

### 5.3. Elispots for Multiplex Analysis

Elispots not only provide superior quantitative performance, they are also amenable to multiplex analyses carried out simultaneously (single well), in parallel wells, or in multi-color assays. Given the relatively small sample size required per test, multiple cytokine Elispots can be performed in parallel. Well established dual and even triple-color Elispots, both enzymatic and fluorescent, allow multiparameter resolution at the single cell level and are currently used in many research settings, including clinical diagnostics as well as therapeutic design [38,39,40]. The ever-expanding availability of discrete fluorochromes, when combined with multi-laser instrumentation and fully automated sample acquisition and data analysis, provide the framework for unsurpassed multi-functional analysis of antigen-specific T cell frequencies in the near future.

### 5.4. Elispots in Limited Resource Settings

Beyond performance criteria, the Elispot assay possesses a number of other characteristics favorable for widespread adoption in developing countries where MTB is most devastating. Compared with flow cytometry, Elispots require one-tenth less cells per test, a fact that is crucial under conditions where samples are precious (remote testing) and/or limiting (pediatric test subjects). Smaller sample sizes also mean less reagents used per test. Also, Elispot data acquisition/analysis is far easier to perform, less time consuming, and dramatically lower in cost than flow cytometry. In fact, data from a 96-well Elispot plate can be acquired and analyzed by an image-based platform, such as the ImmunoSpot, in the same amount of time it takes a skilled flow cytometer operator to collect one sample. Also, while flow cytometry struggles with a lack of user-independent gating capabilities, and therefore suffers from inter-user and inter-lab subjectivity [41], automated platforms promote the standardization of Elispot data analysis and greater reproducibility across sites. These features make the Elispot assay the ideal choice for high-throughput testing applications as could be applied in large-scale subject profiling.

## 6. Conclusions

When assay sensitivity, ease of use in the field, and cost are all taken into consideration, a combination Elispot platform is likely the superior choice for the development of a multifunctional T cell assay to distinguish LTBI from active MTB disease status. Due to the wide variation in cytokine secreting cells per donor within both the LTBI and active MTB subjects [17], we predict that the resolving bio-signatures will be derived from a potentially complex ratio of the frequencies for a number of distinct cytokine–producing T cell types. For this to succeed, the initial enabling phase should be a large scale screen of the *ex vivo* functional profiles from LTBI and active MTB subject groups. To date, one study has compared multi-functional responses (via a Luminex bead array platform) from subject groups containing 12 active TB and 32 LTBIs. Despite the small numbers, the combined use of IL-15 and MCP-1-determined LTBI from active MTB disease states with 88% and 83% accuracy, respectively [29]. Once an accurate panel of distinguishing secreted bio-markers is found, work can begin on converting this assay from cell-based to serum profiling formats, ultimately resulting in the development of rapid remote-applicable testing platforms. 

## References

[1] Kwan C.K., Ernst J.D. (2011). HIV and tuberculosis: A deadly human syndemic. Clin. Microbiol. Rev..

[2] Huynh K.K., Joshi S.A., Brown E.J. (2011). A delicate dance: Host response to mycobacteria. Curr. Opin. Immunol..

[3] Milburn H. (2007). Key issues in the diagnosis and management of tuberculosis. J. R. Soc. Med..

[4] Tu H.A., Vu H.D., Rozenbaum M.H., Woerdenbag H.J., Postma M.J. (2012). A review of the literature on the economics of vaccination against TB. Expert Rev. Vaccines.

[5] Lalvani A., Pareek M. (2010). Interferon gamma release assays: Principles and practice. Enferm. Infecc. Microbiol. Clin..

[6] Diel R., Loddenkemper R., Niemann S., Meywald-Walter K., Nienhaus A. (2011). Negative and positive predictive value of a whole-blood interferon-γ Release assay for developing active tuberculosis: An update. Am. J. Respir. Crit. Care Med..

[7] Ruhwald M., Aabye M.G., Ravn P. (2012). IP-10 release assays in the diagnosis of tuberculosis infection: Current status and future directions. Expert Rev. Mol. Diagn..

[8] Dheda K., van Zyl Smit R., Badri M., Pai M. (2009). T-cell interferon-gamma release assays for the rapid immunodiagnosis of tuberculosis: Clinical utility in high-burden *vs.* low-burden settings. Curr. Opin. Pulm. Med..

[9] Sester U., Fousse M., Dirks J., Mack U., Prasse A., Singh M., Lalvani A., Sester M. (2011). Interferon-gamma release assays for the diagnosis of active tuberculosis: A systematic review and meta-analysis. Eur. Respir. J..

[10] Berry M.P., Graham C.M., McNab F.W., Xu Z., Bloch S.A., Oni T., Wilkinson K.A., Banchereau R., Skinner J., Wilkinson R.J. (2010). An interferon-inducible neutrophil-driven blood transcriptional signature in human tuberculosis. Nature.

[11] Khader S.A., Bell G.K., Pearl J.E., Fountain J.J., Rangel-Moreno J., Cilley G.E., Shen F., Eaton S.M., Gaffen S.L., Swain S.L. (2007). IL-23 and IL-17 in the establishment of protective pulmonary CD4+ T cell responses after vaccination and during Mycobacterium tuberculosis challenge. Nat. Immunol..

[12] Snyder J.E., Bowers W.J., Livingstone A.M., Lee F.E., Federoff H.J., Mosmann T.R. (2003). Measuring the frequency of mouse and human cytotoxic T cells by the Lysispot assay: Independent regulation of cytokine secretion and short-term killing. Nat. Med..

[13] Zajac A.J., Blattman J.N., Murali-Krishna K., Sourdive D.J., Suresh M., Altman J.D., Ahmed R. (1998). Viral immune evasion due to persistence of activated T cells without effector function. J. Exp. Med..

[14] Tigno-Aranjuez J.T., Lehmann P.V., Tary-Lehmann M. (2009). Dissociated induction of cytotoxicity and DTH by CFA and CpG. J. Immunother..

[15] Snyder-Cappione J.E., Divekar A.A., Maupin G.M., Jin X., Demeter L.M., Mosmann T.R. (2006). HIV-specific cytotoxic cell frequencies measured directly ex vivo by the Lysispot assay can be higher or lower than the frequencies of IFN-gamma-secreting cells: Anti-HIV cytotoxicity is not generally impaired relative to other chronic virus responses. J. Immunol..

[16] Lee S.W., Lee C.T., Yim J.J. (2010). Serial interferon-gamma release assays during treatment of active tuberculosis in young adults. BMC Infect. Dis..

[17] Harari A., Rozot V., Enders F.B., Perreau M., Stalder J.M., Nicod L.P., Cavassini M., Calandra T., Blanchet C.L., Jaton K. (2011). Dominant TNF-alpha+ Mycobacterium tuberculosis-specific CD4+ T cell responses discriminate between latent infection and active disease. Nat. Med..

[18] Addo M.M., Yu X.G., Rathod A., Cohen D., Eldridge R.L., Strick D., Johnston M.N., Corcoran C., Wurcel A.G., Fitzpatrick C.A. (2003). Comprehensive epitope analysis of human immunodeficiency virus type 1 (HIV-1)-specific T-cell responses directed against the entire expressed HIV-1 genome demonstrate broadly directed responses, but no correlation to viral load. J. Virol..

[19] Day C.L., Abrahams D.A., Lerumo L., Janse van Rensburg E., Stone L., O'Rie T., Pienaar B., de Kock M., Kaplan G., Mahomed H. (2011). Functional capacity of Mycobacterium tuberculosis-specific T cell responses in humans is associated with mycobacterial load. J. Immunol..

[20] Casey R., Blumenkrantz D., Millington K., Montamat-Sicotte D., Kon O.M., Wickremasinghe M., Bremang S., Magtoto M., Sridhar S., Connell D. (2010). Enumeration of functional T-cell subsets by fluorescence-immunospot defines signatures of pathogen burden in tuberculosis. PLoS One.

[21] Betts M.R., Nason M.C., West S.M., De Rosa S.C., Migueles S.A., Abraham J., Lederman M.M., Benito J.M., Goepfert P.A., Connors M. (2006). HIV nonprogressors preferentially maintain highly functional HIV-specific CD8+ T cells. Blood.

[22] Harari A., Dutoit V., Cellerai C., Bart P.A., Du Pasquier R.A., Pantaleo G. (2006). Functional signatures of protective antiviral T-cell immunity in human virus infections. Immunol. Rev..

[23] Sester U., Fousse M., Dirks J., Mack U., Prasse A., Singh M., Lalvani A., Sester M. (2011). Whole-blood flow-cytometric analysis of antigen-specific CD4 T-cell cytokine profiles distinguishes active tuberculosis from non-active states. PLoS One.

[24] Caccamo N., Guggino G., Joosten S.A., Gelsomino G., Di Carlo P., Titone L., Galati D., Bocchino M., Matarese A., Salerno A. (2010). Multifunctional CD4(+) T cells correlate with active Mycobacterium tuberculosis infection. Eur. J. Immunol..

[25] Chen X., Zhang M., Liao M., Graner M.W., Wu C., Yang Q., Liu H., Zhou B. (2010). Reduced Th17 response in patients with tuberculosis correlates with IL-6R expression on CD4+ T cells. Am. J. Respir. Crit. Care Med..

[26] Scriba T.J., Kalsdorf B., Abrahams D.A., Isaacs F., Hofmeister J., Black G., Hassan H.Y., Wilkinson R.J., Walzl G., Gelderbloem S.J. (2008). Distinct, specific IL-17- and IL-22-producing CD4+ T cell subsets contribute to the human anti-mycobacterial immune response. J. Immunol..

[27] Li L., Qiao D., Fu X., Lao S., Zhang X., Wu C. (2011). Identification of Mycobacterium tuberculosis-specific Th1, Th17 and Th22 cells using the expression of CD40L in tuberculous pleurisy. PLoS One.

[28] Hirsch C.S., Toossi Z., Othieno C., Johnson J.L., Schwander S.K., Robertson S., Wallis R.S., Edmonds K., Okwera A., Mugerwa R. (1999). Depressed T-cell interferon-gamma responses in pulmonary tuberculosis: Analysis of underlying mechanisms and modulation with therapy. J. Infect. Dis..

[29] Frahm M., Goswami N.D., Owzar K., Hecker E., Mosher A., Cadogan E., Nahid P., Ferrari G., Stout J.E. (2011). Discriminating between latent and active tuberculosis with multiple biomarker responses. Tuberculosis (Edinb).

[30] Ellner J.J. (2010). Immunoregulation in TB: Observations and implications. Clin. Transl. Sci..

[31] Hirsch C.S., Hussain R., Toossi Z., Dawood G., Shahid F., Ellner J.J. (1996). Cross-modulation by transforming growth factor beta in human tuberculosis: Suppression of antigen-driven blastogenesis and interferon gamma production. Proc. Natl. Acad. Sci. USA.

[32] Harris J., De Haro S.A., Master S.S., Keane J., Roberts E.A., Delgado M., Deretic V. (2007). T helper 2cytokines inhibit autophagic control of intracellular Mycobacterium tuberculosis. Immunity.

[33] Streeck H., Frahm N., Walker B.D. (2009). The role of IFN-gamma Elispot assay in HIV vaccine research. Nat. Protoc..

[34] Hesse M.D., Karulin A.Y., Boehm B.O., Lehmann P.V., Tary-Lehmann M. (2001). A T cell clone’s avidity is a function of its activation state. J. Immunol..

[35] Zhang W., Caspell R., Karulin A.Y., Ahmad M., Haicheur N., Abdelsalam A., Johannesen K., Vignard V., Dudzik P., Georgakopoulou K. (2009). ELISPOT assays provide reproducible results among different laboratories for T-cell immune monitoring—Even in hands of ELISPOT-inexperienced investigators. J. Immunotoxicol..

[36] Lehmann P.V., Zhang W. (2012). Unique strengths of ELISPOT for T cell diagnostics. Methods Mol. Biol..

[37] Lash G.E., Pinto L.A. (2010). Multiplex cytokine analysis technologies. Expert Rev. Vaccines.

[38] Karulin A.Y., Hesse M.D., Tary-Lehmann M., Lehmann P.V. (2000). Single-cytokine-producing CD4 memory cells predominate in type 1 and type 2 immunity. J. Immunol..

[39] Quast S., Zhang W., Shive C., Kovalovski D., Ott P.A., Herzog B.A., Boehm B.O., Tary-Lehmann M., Karulin A.Y., Lehmann P.V. (2005). IL-2 absorption affects IFN-gamma and IL-5, but not IL-4 producing memory T cells in double color cytokine ELISPOT assays. Cell. Immunol..

[40] Rebhahn J.A., Bishop C., Divekar A.A., Jiminez-Garcia K., Kobie J.J., Lee F.E., Maupin G.M., Snyder-Cappione J.E., Zaiss D.M., Mosmann T.R. (2008). Automated analysis of two- and three-color fluorescent Elispot (Fluorospot) assays for cytokine secretion. Comput. Methods Programs Biomed..

[41] Janetzki S., Britten C.M., Kalos M., Levitsky H.I., Maecker H.T., Melief C.J., Old L.J., Romero P., Hoos A., Davis M.M. (2009). “MIATA”-minimal information about T cell assays. Immunity.

